# Clinical and epidemiological profiles of patients with American cutaneous leishmaniasis from the states of Pernambuco and Amazonas, Brazil

**DOI:** 10.1590/0037-8682-0083-2020

**Published:** 2020-11-25

**Authors:** Maria Gabriella Nunes de Melo, Rayana Carla Silva de Morais, Tayná Correia de Goes, Rômulo Pessoa e Silva, Rômulo Freire de Morais, Jorge Augusto de Oliveira Guerra, Maria Edileuza Felinto de Brito, Sinval Pinto Brandão, Milena de Paiva Cavalcanti

**Affiliations:** 1 Fundação Oswaldo Cruz, Instituto Aggeu Magalhães, Recife, PE, Brasil.; 2 Fundação de Medicina Tropical Dr. Heitor Vieira Dourado, Manaus, AM, Brasil.

**Keywords:** American cutaneous leishmaniasis, Clinical profile, Epidemiology, Surveillance

## Abstract

**INTRODUCTION:**

Brazil has a high number of cases of American cutaneous leishmaniasis (ACL) in the north and northeast regions. Therefore, continuous surveillance of environmental and socioeconomic factors in endemic areas is needed to develop strategic control measures. This study aimed to describe the clinical and epidemiological profiles of patients with ACL.

**METHODS:**

All patients were from the states of Amazonas and Pernambuco, and examinations were carried out between 2015 and 2018. All patients had a clinical and epidemiological history compatible with ACL after positive diagnostic tests. Information obtained from medical records included gender, employment activity, level of education, age, and number and sites of lesions.

**RESULTS:**

A total of 213 patients were included, of whom 30.98% were female and 69.02% were male. The main employment activity was agriculture (27.56%). The most common level of education was elementary (62.42%). The average age was approximately 39 years. The majority of the patients presented only with one lesion (54.87%), and legs/feet were the most commonly affected area (48.25%), followed by the arms/hands (44.75%).

**CONCLUSIONS:**

These data demonstrated that irrespective of the patients’ places of origin, interventions need to be focused on men of economically productive age, in view of the high risk of exposure to the vector in this group. Education activities need to be directed to farmers about the importance of protection against ACL vectors during work. Such information must also be directed to employers as a way of implementing and maintaining appropriate working conditions and stepping up vector control.

## INTRODUCTION

According to the World Health Organization (WHO), leishmaniasis is one of the seven most important tropical diseases in the world^1^. Cutaneous leishmaniasis (CL) is a zoonosis caused by different species of protozoa belonging to the genus *Leishmania* (Order: Kinetoplastida, Family: Trypanosomatidae)^2^, which are intracellular parasites of the mononuclear phagocytic system and are transmitted to humans and wild or domestic animals through the bite of infected sand flies of the genus *Phlebotomus* (Old World) or *Lutzomyia* (New World)^1^ ^,^ ^3^ ^,^ ^4^.

CL is widely prevalent and possesses specific epidemiological characteristics in the New World, where the disease is known as American cutaneous leishmaniasis (ACL)^5^. Cases have been reported in countries lying between the southern United States and northern Argentina, with the exception of Chile, Uruguay, and El Salvador. Eleven dermotropic species of this disease have been identified, with eight belonging to the *Viannia* subgenus*,* and three to *Leishmania* ^1^. ACL is characterized as a pool of diseases with distinct clinical and immunopathological manifestations, in which the development and exacerbation of host symptoms are related to a variety of factors such as the parasite species involved in the infection, immunological and/or nutritional status of the patient, age of the patient, and whether the patient resides in an endemic area^6^. 

ACL was initially classified as an enzootic disease of wild animals. However, with increased deforestation for the expansion of urban centers and the construction of roads for commercial purposes, the disease has become zoonotic. Humans are exposed to the risk of infection through their relationship with other hosts, and this may be related to social behavior, beliefs, regional habits, family traditions, work and leisure activities, and ecological factors (such as climate and environmental preservation). Thus, the most socioeconomically disadvantaged populations are the most affected by the disease^7^ ^-^ ^9^, although it should be noted that the distribution of leishmaniasis depends on the presence of insect vectors and the movement of reservoir mammals^12^ ^,^ ^13^.

According to WHO data, in 2017, the majority of ACL cases reported worldwide occurred in Afghanistan, Algeria, Brazil, Colombia, the Islamic Republic of Iran, Pakistan, Peru, Saudi Arabia, and the Syrian Arab Republic^8^. In Brazil, analysis of the chronology of the disease from 2007 to 2017 (the latest data) shows that ACL is currently present in all the Brazilian states in all the five regions, with a total of 235,301 reported cases. The north and northeast regions are the most affected, with 101,332 and 72,395 reported cases, respectively. The states of Pará and Amazonas in the north account for 58% of all cases.

In the northeast, the states of Maranhão, Ceará, Bahia, and Pernambuco present with a significant number of reported cases, accounting for 95.73% (69,306) of all cases in this region^10^. According to the epidemiological bulletin produced by the Health Surveillance Department in Brazil, more than 300,000 cases of ACL were recorded between 2003 and 2018, with an annual average of 21,158. The north region had the highest number of cases during this period^14^.

In ACL, the parasite affects the lining of the epithelial tissue, leading to cutaneous lesions. However, depending on the etiological species, the parasite may access the hematogenous pathway and damage regions of the mucosal upper respiratory tract such as the nose, pharynx, and larynx. Clinical manifestations can be classified as localized, disseminated, diffuse, or mucocutaneous^11^.

Diagnosis of leishmaniasis is based on criteria involving epidemiological, clinical, and laboratory data^15^ ^,^ ^16^. The reliability and speed of the final diagnosis is pivotal for ensuring rapid appropriate treatment of the patient and establishing important intervention strategies for the control of this disease^17^. Currently, there is neither an effective, accessible, and safe treatment strategy nor an approved vaccine against ACL, underlining the need to develop new clinical-therapeutic strategies^18^. 

In the north region of Brazil, the state of Amazonas (AM) has an especially high incidence of cases^10^ and transmission, which is often related to employment, vegetable extraction activities, disorderly deforestation, and forest leisure programs. Due to these activities, humans expose themselves to the wild cycle of the disease and become infected^19^. In the northeast region, the state of Pernambuco (PE) has a high number of reported cases among rural workers, who are constantly exposed to endemic localities and risk factors^20^. However, changes can be seen in clinical, epidemiological, and therapeutic management. These can differ from one region to another because of vector diversity, reservoirs, etiological agents, socioeconomic and environmental conditions, and knowledge of the disease^21^. Therefore, constant surveillance of endemic areas concerning environmental, economic, and social features is needed. This study aimed to describe the clinical and epidemiological profiles of patients from areas endemic for ACL in the states of Amazonas and Pernambuco and to highlight differences and similarities, thereby helping to expand the knowledge required for the development of effective strategic control programs in each region studied. 

## METHODS

### Study area

**FIGURE 1:**
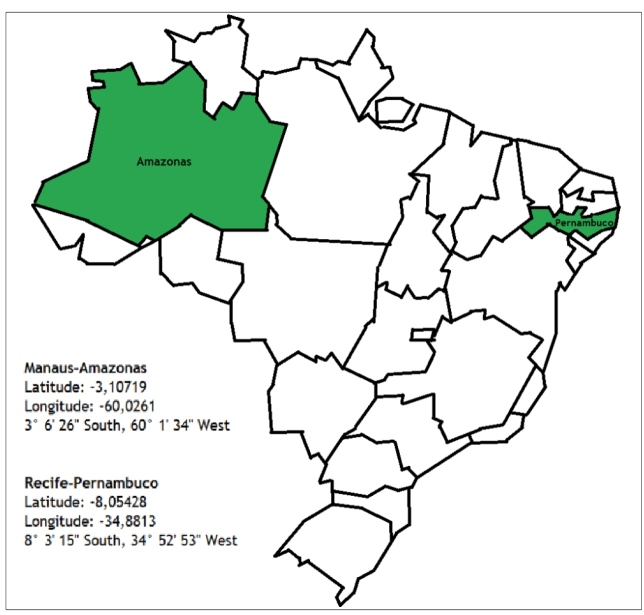
Map of Brazil and states from which patients were included for the comparative study of American cutaneous leishmaniasis. **Source:** NicePNG (adapted by the author).

This was a descriptive study of data provided by patients with ACL. Convenience sampling was adopted^22^, with the samples being chosen from the Dr. Heitor Vieira Dourado Foundation of Tropical Medicine in the state of Amazonas (HVD-FTM/AM) located in the north region of Brazil, and from the Leishmaniasis Referral Service, Oswaldo Cruz Foundation, in the state of Pernambuco (LRS-FIOCRUZ/PE) in the northeast region. Additionally, some of the patients included were from active searches performed between 2015 and 2018 through partnerships with the Municipal Health Department of Igarassu and Cabo de Santo Agostinho, Pernambuco. Figure 1 shows both the Brazilian states. 

### Diagnosis and collection of clinical-epidemiological data

According to the convenience of the HVD-FTM/AM, LRS-FIOCRUZ/PE, and the Municipal Health Department of Igarassu and Cabo de Santo Agostinho - Pernambuco, individuals with a clinical (presence of active lesion) and epidemiological history (living and/or working in areas where ACL is endemic) compatible with ACL, and one or more positive diagnostic tests were included in the study. To determine the diagnosis, direct search test and/or parasitological isolation in culture, conventional polymerase chain reaction (PCR)^22^, and quantitative real-time PCR (qPCR)^23^ were performed. 

All the patients underwent direct examinations; the smear test was carried out by scarifying the inner edge of the ulcer or the surface of the closed skin lesion using sterile scalpel blades. The smear was then stained and analyzed by optical microscopy to search for amastigote forms of the parasite. During parasitological isolation in culture, a biopsy of the ulcer border or lesion aspirate was inoculated into culture media with modified blood agar and kept at 24-26 °C. After the fifth day, in the positive samples, promastigote forms of the parasite could already be found.

The molecular diagnosis was determined through conventional PCR using the B1B2 system, which amplifies the kDNA target^23^, and qPCR, which amplifies the kDNA target^24^. Both techniques were performed according to the instructions of their developers^23^ ^,^ ^24^.

**FIGURE 2:**
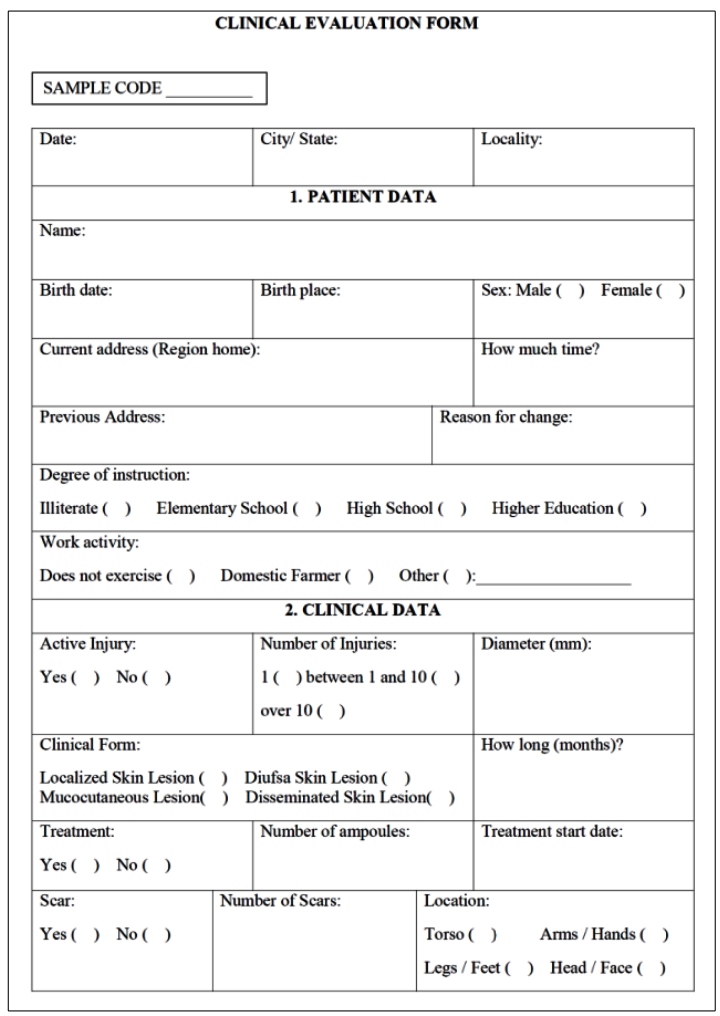
Individual survey with clinical and epidemiological data.

All the diagnostic tests were performed by the health services that the patients attended. During sample collection, an individual form with items related to clinical and epidemiological data was completed by each patient (Figure 2).

### Ethical considerations

The present study was approved by the research and ethics committee of the Aggeu Magalhães Institute (AMI-FIOCRUZ/PE) and HVD-FTM/AM. All the participants signed the Term of Free Informed Consent.

### Data analysis

The data contained in the forms were used to perform a quantitative analysis of gender, age, number of lesions, lesion site, employment activity, and level of education. Statistical analysis was performed using descriptive statistics, with absolute figures and percentages. 

## RESULTS

A total of 213 patients participated in this study, 92 of them were from the state of Pernambuco and 121 were from the state of Amazonas. All patients had a clinical (presence of active lesion) and epidemiological history (living and/or working in areas where ACL is endemic) compatible with ACL, and one or more positive diagnostic tests. All the patients presented with localized cutaneous leishmaniasis (active skin lesion). The durations of illness varied according to the patient, with an average of 116 days for the patients in Pernambuco and 37 days for those in Amazonas. Sixty-six of these patients were female (37 PE; 29 AM) (30.98%) and 147 (55 PE; 92 AM) (69.02%) were males. The youngest patient was 11 years old, while the oldest was 73. The average age was approximately 39 years, which is an economically productive age.

**FIGURE 3:**
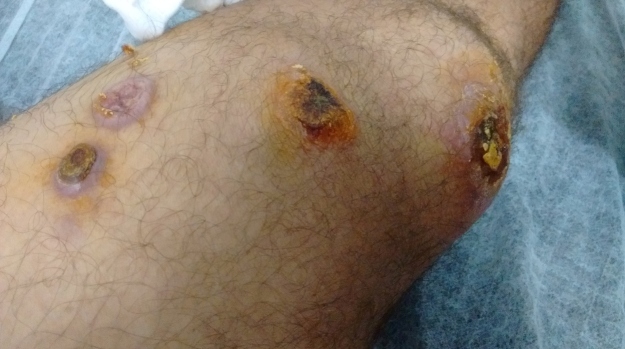
A patient from Amazonas who presented with multiple lesions in the lower limbs.

**FIGURE 4:**
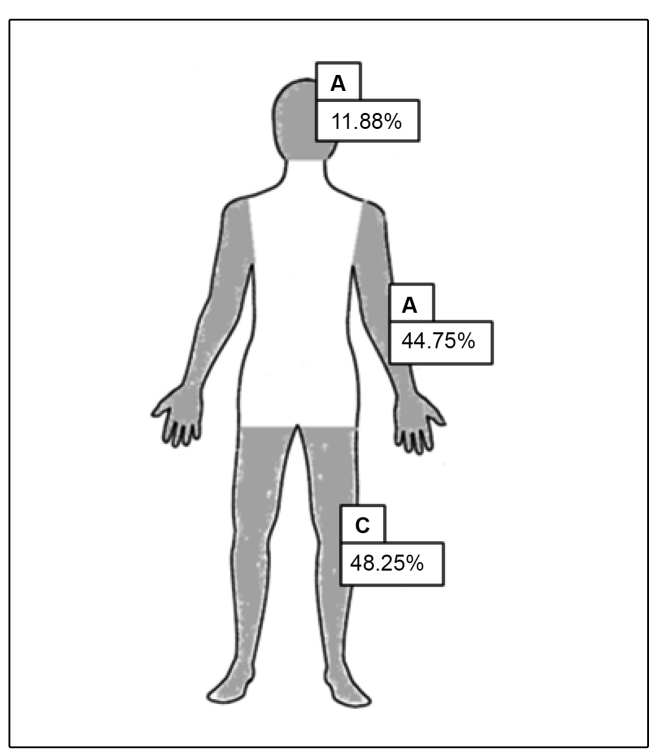
Percentages for body sites that were most affected by lesions. **A:** Head; **B:** Arms/hands; **C:** Legs/feet.

The number of lesions was reported in 195 patients (74 PE, 121 AM). Considering the total number of individuals regardless of origin, the majority presented with only one lesion (54.87%) (54 PE; 53 AM), followed by presentation of more than one lesion (41.54%) (18 PE; 63 AM), and over 10 lesions (3.59%) (2 PE; 5 AM). In Pernambuco, a higher number of individuals with only one lesion (27.7%) was found compared with Amazonas, which reported a higher percentage of patients with more than one lesion (34.87%). The affected body sites were described in 143 patients, and an analysis of this demonstrated that the legs/feet were the most affected region (n = 69, 48.25%) (Figure 3). The arms/hands were the second-most affected region (n = 64, 44.75%), followed by the head (n = 17, 11.88%) (Figure 4) (Table 1).

**TABLE 1:** Quantitative analysis of gender, number/sites of lesions, employment activity, and level of education presented in clinical reports of patients attended during 2015-2018 in the states of Pernambuco and Amazonas.

Variables	Total	PE	AM
**Gender (n=213)**			
Female	66	37	29
Male	147	55	92
**Number of lesions (n=195)**			
One lesion	107	54	53
More than one lesion	81	18	63
Over 10 lesions	7	2	5
**Body sites affected by lesions (n=143)**			
Arms/Hands	64	11	53
Legs/Feet	69	29	40
Head	17	3	14
**Employment activity (n=185)**			
Agriculture	51	16	35
Construction	18	0	18
Study	16	15	1
**Level of Education (n=165)**			
Full elementary education	72	28	44
Incomplete elementary education	31	1	30
High school	38	6	32
Higher education	14	6	8
Illiterate	10	5	5

**PE:** Pernambuco; **AM:** Amazonas; **n:** number of patients who provided data.

One hundred and eighty-five patients provided data on employment. Agricultural activities were common in both the states (n = 51, 27.56%) (16 PE; 35 AM), followed by construction (n = 18, 9.72%) (all 18 from AM), and study (n = 16, 8.65%) (15 PE; 1 AM). One hundred and sixty-five patients reported data on level of education: 103 had elementary education (62.42%) (29 PE; 74 AM), 72 of whom had completed this level (69.9%) (28 PE; 44 AM) and 31 of whom did not complete elementary education (30.1%) (1 PE; 30 AM), 38 completed high school (23.03%) (6 PE; 32 AM), 14 completed higher education (8.48%) (6 PE; 8 AM), and 10 were illiterate (6.06%) (5 PE; 5 AM).

## DISCUSSION

The current study performed a clinical-epidemiological analysis of patients with ACL from the states of Pernambuco and Amazonas located in the northeast and north regions of Brazil, respectively. It was observed that individuals within the economically productive age group and males were the most affected in both the states. Overall, agriculture was the main employment activity of the ACL patients; although in Pernambuco, a large number of infected students were noted, and in Amazonas, a significant proportion of cases occurred among construction workers. 

The individuals in this study had an average age of approximately 39 years, which lies within the economically productive range. In line with our finding, a study by Grangeiro-Júnior et al.^25^ in Ceará, northeast Brazil found that 56.47% of male and 43.53% of female patients had an average age of 34.7 years. According to a study by Oliveira et al.^26^ in Jussara/Paraná, those aged between 20 and 39 years were the most affected. In Argentina, Bustos et al.^27^ reported that the majority of patients (78.9%) were male, with an average age of 39.1 years. Therefore, it has been found that ACL affects mainly economically productive males, regardless of etiological and epidemiological differences. 

A high prevalence of ACL in adult men (reproductive phase) has been reported in various studies^28^ ^-^ ^30^. These studies also found that the disease affects women and children from the same family. Brito et al.^20^ and Oliart-guzmán et al.^30^ found that most of the individuals affected by ACL were males living in rural areas. These previous studies from the states of Pernambuco and Amazonas corroborate our findings. A study conducted by Grangeiro-Júnior et al.^25^ in Ceará found that most of the patients (89.53%) were from rural areas and that the main activities for both genders were agriculture and study, thus, corroborating our results for Pernambuco. This pattern was found in both the states covered in our study. Furthermore, in peri-urban and rural areas, transmission may occur in schools and domestic environments, which could in turn affect individuals of both genders and of different ages, as observed in Pernambuco. According to a study by Medina-Morales, Machado-Duque, and Machado-Alba^31^, the high frequency of transmission in students (80% of the patients) in the municipality of Pueblo Rico in Risaralda-Colombia may be linked to the concentration of vectors in this area, which lies in the vicinity of the forest. This may explain the similar situation in Pernambuco, since most of the patients were from rural areas. 

A study conducted by Teles et al.^32^ in the states of Acre and Amazonas found a high percentage of male individuals (81.1%) from rural areas (75.7%), suggesting that cases of ACL are linked to agricultural activities in a forest environment and that transmission may occur outside of the home during working hours. These results are confirmed by our study, since most of the patients in Amazonas were farmers, and some of them were construction workers. While carrying out both the activities, individuals are susceptible to infection. Exposure to the vector is more frequent in such professions, especially among those working in agriculture and those living in rural and peri-urban regions, owing to the contact with the wild environment and deforested areas^5^. Employment activities such as construction can also modify the vector’s natural habitat and thus, can cause infection as a result of stress and adaptation of the vector to new environments^33^.

Most of the patients included in the current study had only one lesion. Consistent with this finding, Figueira et al.^34^ demonstrated that in the municipality of Rio Preto da Eva in the state of Amazonas, at least 56.7% of the individuals with ACL presented with only one lesion and 43.4% presented with more than one. In a study conducted by Castro et al.^35^ in the north of Paraná, 67% of the individuals were found to have only one lesion and 31% had two or more. Some of these studies were conducted in or near the state of Amazonas. The clinical manifestation characterized by the occurrence of more than one lesion in patients in Amazonas may be related to multiple bites by infected sand flies, metastatic lymphatic spreading^36^, or even the etiological diversity existing in the state. However, most of the infected patients (in Pernambuco and Amazonas) presented with only one lesion. This may be related to the species that caused the infection. 

In Amazonas, it is known that *Leishmania guyanensis* is the species that is mainly responsible for cases of ACL. Its principal vector is *Lu*. *Umbratilis* phleobotomine^30^ ^,^ ^37^, which is found in the early hours of the morning in moist soil environments such as the trunks of large trees^38^. One unique feature of phlebotomine is the high number of bites it causes in individuals. In a study conducted by Gomes et al.^39^ in Amazonas, *Lu*. *umbratilis* was found in large numbers in military training grounds in the Amazon rainforest.

It is known that the infection caused by *Leishmania* spp. is related to patterns of exploitation of land, occupation, and construction in endemic areas. Most of the phlebotomine species found in the regions covered by the current study exhibited daytime blood-feeding habits. This coincides with patients’ employment activities and may therefore, be the cause of single or multiple ulcerative cutaneous lesions^40^. As the timing of occupational activities coincides with the vector’s biological cycle, another factor that may influence the number of lesions is the type and place of work of the patients. Thus, the current study showed that the largest number of farmers who presented with more than one lesions was found in the state of Amazonas. 

In Pernambuco, it was found that the body sites that were most affected were the lower limbs, whereas in Amazonas, the upper limbs were the most affected. These data are linked to habits and to the body sites that are most exposed to phlebotomine bites, as observed in studies by Paniz-Mondolfi et al.^5^and Brito et al.^41^, which showed that the most exposed areas and those most susceptible to the vector in a clinically localized form are the face, upper limbs, and lower limbs. Castro et al.^34^ showed that cutaneous lesions were located primarily in the lower limbs (47.7%), followed by the upper limbs (26.7%), and the face (16.0%), corroborating the results of our study. Grangeiro-Júnior et al.^25^ reported that the lower limbs were the most affected body site (n = 164, 45.18%), followed by the upper limbs (n = 82, 22.59%), head (n = 34, 9.37%), and abdomen (n = 24, 6.61%). Considering the size of the lesions and body sites affected, these injuries may interfere with the patient’s quality of life and may cause disfigurement, which may in turn harm both familial and social interpersonal relationships, and may also lead to psychosocial disorders such as depression^42^ ^,^ ^43^. 

Regarding the level of education, the present study found that most of the patients in both the states had elementary education (62.42%), while a minority were illiterate (6.06%), corroborating the findings of a study by Vasconcelos, Araújo, and Rocha^28^, which found that 79.3% of individuals with ACL had incomplete elementary education levels (n = 119) and 10.7% were illiterate (n = 16). Their study was conducted among patients living in the rural parts of Pernambuco. Similarly, a study performed by Oliveira et al.^48^ showed that 49% of individuals had incomplete elementary schooling. In a study by Oliveira et al.^26^, performed in the municipality of Jussara-Paraná, reported that 68% (n = 212) of the participants had attended school for only seven years. In a study by Graziani, Oliveira, and Silva^44^ in Goiás, it was found that among individuals affected by ACL, 56.0% (n = 724) had incomplete elementary education, 11.6% (n = 142) had completed elementary education, and only 6.6% (n = 86) had completed high school. Owing to the poor accessibility and availability of schools, most of the farmers who lived in rural areas did not have a high level of education. The lack of education and access to information, in combination with low income in most cases, hinder the work of health services^45^.

All these factors help in perpetuating the endemic status of numerous diseases that affect rural areas. The majority of individuals affected by ACL live in different settings^46^. Poor housing conditions and lack of basic sanitation mean that patients are usually unaware of protection and control measures regarding infected sand flies^47^. Environmental and socioeconomic factors may determine the clinical course and outcomes of treatment, since living conditions and lack of transportation may be associated with difficulties in accessing diagnostic tests and continuous treatment. Other factors such as low income and low levels of education, in combination with poor nutrition and other infectious diseases, may also hamper efforts to achieve clinical management and may contribute to a more severe disease course^48^ ^,^ ^49^. According to Alvar, Yactayo, and Bern^47^, numerous factors linked to poverty may play a role in the development of leishmaniasis and concurrent diseases. These include living conditions, malnutrition, environmental sanitation, lack of individual protection measures, type of work, and deforestation. 

The results of this study provided a basis for understanding the current epidemiological situation of the study population. This may enable the proper control and management of ACL by stepping up preventive action and implementing policy control, taking into account the disease features specific to each area covered by the study. One suggestion involves the creation of a plan that includes effective early diagnosis and treatment of leishmaniasis. Provision of proper medical assistance for patients and management through surveillance would also be an effective strategy for the prevention and control of the disease. 

The data obtained in this study indicated that regardless of the patients’ places of origin (rural or urban), action should be focused on males of economically productive age. The lesion time was shorter in patients from Amazonas, possibly due to the greater access to healthcare and greater commitment to treatment. Furthermore, there was a difference in the number of lesions between patients in each state; in Pernambuco, patients predominantly presented with one lesion, while in Amazonas, patients presented with more than one lesion. This might be due to *Leishmania* and vector species present in each region, considering the voracity of *Lu. umbratilis* in Amazonas. In addition, health education activities in both the states should be developed using dynamic information. This can help prevent and control the disease and increase awareness in the affected population. Some insect vector control measures need to be related to the habits of each species. Exposure to the vector can also be avoided by encouraging the continuous use of insecticides, protective screens on windows, and fine-mesh mosquito nets. The current study showed that difficulties in eliminating ACL and its clinical symptoms may be related to the socioeconomic disadvantages of the affected populations. In view of this, it is important to develop an innovative health education program for farmers that demonstrates the importance of individual protection during work and thus, helps to improve the living conditions, working conditions, and health of the affected population.
